# Variants of a *Thermus aquaticus* DNA Polymerase with Increased Selectivity for Applications in Allele- and Methylation-Specific Amplification

**DOI:** 10.1371/journal.pone.0096640

**Published:** 2014-05-06

**Authors:** Matthias Drum, Ramon Kranaster, Christina Ewald, Rainer Blasczyk, Andreas Marx

**Affiliations:** 1 Department of Chemistry, Konstanz Research School Chemical Biology, University of Konstanz, Konstanz, Germany; 2 myPOLS Biotec, University of Konstanz, Konstanz, Germany; 3 Institute for Transfusion Medicine, Hannover Medical School, Hannover, Germany; The Scripps Research Institute, United States of America

## Abstract

The selectivity of DNA polymerases is crucial for many applications. For example, high discrimination between the extension of matched versus mismatched primer termini is desired for the detection of a single nucleotide variation at a particular locus within the genome. Here we describe the generation of thermostable mutants of the large fragment of *Thermus aquaticus* DNA polymerase (*KlenTaq*) with increased mismatch extension selectivity. In contrast to previously reported much less active *KlenTaq* mutants with mismatch discrimination abilities, many of the herein discovered mutants show conserved wild-type-like high activities. We demonstrate for one mutant containing the single amino acid exchange R660V the suitability for application in allele-specific amplifications directly from whole blood without prior sample purification. Also the suitability of the mutant for methylation specific amplification in the diagnostics of 5-methyl cytosines is demonstrated. Furthermore, the identified mutant supersedes other commercially available enzymes in human leukocyte antigen (HLA) analysis by sequence-specific primed polymerase chain reactions (PCRs).

## Introduction

Personalized medicine providing therapies, adapted to each patient's genetic predisposition[1] [2] [3] [4] [5], is mainly supported by the analysis of single nucleotide polymorphisms (SNPs)[1]. SNPs are single base variations and besides copy number variations the most abundant type of genetic variation found between members of one species[6] [7] [8] [9]. SNPs located in coding sequences can lead to structural and functional changes in the affected proteins, enzymes or receptors. For example the prothrombin G20210A mutation is one of the most common genetic polymorphisms known to predispose to a first episode of venous thromboembolism[10]. Most SNPs, however, are found in non-coding intergenic regions and often show no phenotypic effect. Intergenic SNPs present interesting markers for the determination of parentage[11] [12], anthropology[13] [14] or forensic tasks e.g. genetic fingerprinting. Many of these variations can affect predispositions for diseases or responses to drugs, chemicals and vaccines[1] [15], which makes them especially interesting for pharmacogenomics[16] [17]. The human genome project greatly contributed to personalized medicine by identifying more than 2.4 million SNPs in 2001[18] [9]. This created a basis for the first human haplotype map (HapMap) project with more than one million SNPs for which accurate and complete genotypes have been obtained in 269 DNA samples from four populations[19]. In a second step an additional 2.1 million SNPs were added[20]. The next phase could further improve the quality with an extended set of 1,184 samples from 11 populations[21]. With the 1000 Genomes Project, a validated haplotype map of 38 million SNPs was published in 2012[22]. Genomes of 1,092 individuals sampled from 14 populations drawn from Europe, East Asia, sub-Saharan Africa and the Americas were analysed through a combination of low-coverage whole-genome sequence data, targeted deep exome sequence data and dense SNP genotype data[22]. It is most likely that SNP genotyping will be one of the future key technologies to diagnose these genetic variations among whole populations as well as in single patients. Different techniques can be used for the analysis of SNPs such as selective primer extension, e.g. minisequencing[23] [24] [25], pyrosequencing[26] or allele specific amplification (ASA)[27] [23] [28]. ASA and selective primer extension[23] depend on the inefficient extension of a mismatched primer/template complex. Therefore, highly selective DNA polymerases are urgently needed. Allele specific amplification through real-time PCR (ASA) allows detection of SNPs in a very efficient way. ASA, unlike most other methods for SNP detection, does not require preliminary amplification of the target genetic material[29] [30]. It combines amplification and detection in a single reaction, based on the discrimination of matched and mismatched primer/template complexes. The increase of amplified DNA during the reaction can be monitored in real-time through the increase of a fluorescence signal caused by a dye such as SYBR Green I[31] emitting upon binding to double-stranded DNA. The match case comprises a correct Watson-Crick base pair at the 3′-primer end, whereas the mismatch case features a non-canonical base pair. The mismatch should result in a less efficient or, ideally, no product amplification[30] [32] [33] [34] [35] [36] [37]. In real-time ASA this is reflected with a delayed or absent fluorescence signal for the mismatch case. In SNP diagnostics this provides information about the absence or presence of an SNP.

Also in transfusion medicine SNP detection is of crucial interest. Human leukocyte antigen (HLA) genes exhibit an unprecedented genetic diversity. PCR-based methods have therefore emerged as the most important techniques for HLA typing[38]. Due to the necessity to consider the cis/trans phases of nucleotide variations in HLA alleles the amplification of SNPs has become a standard procedure in molecular HLA typing. One of the most widely used PCR-based methods for HLA class I and class II typing is the sequence-specific primed PCR (PCR-SSP)[38]. Identification of the HLA type requires a large panel of specific PCR mixes which are run simultaneously using the genomic DNA of a single individual[39]. Each PCR is designed to identify a particular HLA specificity or group of HLA specificities. Although PCR fragments between the different specificities vary in size considerably, the main basis for allele assignment is simply the presence or absence of the PCR products. Thus, a highly selective DNA polymerase is of utmost importance for high quality HLA typing results.

Another area of relevance for highly specific DNA polymerases is methylation-specific PCR (MSP). MSP is a widely used method for the analysis of methylation patterns of cytosines at the C-5 position - the most abundant DNA modification in vertebrates. 5-Methylcytosine (5mC) is an important epigenetic mark and plays a crucial role in activating or silencing genes[40] [41] [42]. Interestingly methylation patterns alter during development and carcinogenesis[43]. Alterations of epigenetic marks like DNA methylation play a crucial role in the onset of diseases like cancer and are therefore important early stage cancer biomarkers[44] [42] [45] [46] [47]. In general, it appears that the detection of epigenetic alterations is a promising and emerging tool for cancer diagnostics, prognostics, and the prediction of response to therapies awaiting broad application in the future. In combination with genetic mutation analysis, epigenetic analysis will result in a very powerful approach along these lines for early diagnostics of cancer and other diseases such as neuro-developmental and metabolic disorders as well as auto-immune diseases[45] [46] [47]. Several methods for the detection of DNA methylation alterations are currently known and employed. The most common approaches to obtain single base resolution are based on ‘bisulfite treatment’ of the genomic DNA sample. By bisulfite treatment cytosine (C) is converted to uracil (U), while 5mC remains unchanged[48] [49]. In MSP bisulfite treated DNA is amplified by PCR using primer pairs spanning the CpG site of interest. Herein, a primer pair is either designed for methylated-specific DNA or unmethylated-specific DNA. Due to the conversion of C into U, mismatches are introduced preventing or enabling efficient PCR amplification, depending on the chosen primer pair[50].

MSP as well as ASA are very cost-effective methods that do not require specialized equipment and can be performed in almost any laboratory. Selectivity is the most important factor in ASA and MSP and can be increased by the use of modified primer probes[51] [52] [53] or by the employment of mutated DNA polymerases possessing a higher mismatch extension selectivity as compared to the wild type enzyme. A selective polymerase would enable reliable ASA and MSP without the need of any substrate modifications and can thus be the most cost and work efficient solution. Several DNA polymerase variants with increased DNA replication fidelity are known[54], e.g. for the Klenow fragment of *E. coli* DNA polymerase I and the thermostable *Thermus aquaticus* (Taq) DNA polymerase[55] or the *Pyrococcus furiosus* (Pfu) DNA polymerase[56]. It is known that the exchange of amino acids, affecting the interaction between polymerase and primer/template complex or the binding pocket's geometry, can lead to a change in selectivity of DNA polymerases[57]. In previous studies, an increase in Taq DNA polymerase selectivity was achieved by substituting the polar amino acid side chains in Q and H (Gln, and His) of motif C for leucine bearing a non-polar side chain. Motif C is highly conserved in the palm domain within DNA polymerase families A, B, X and Y and plays a role in the identification of mismatched bases in the primer/template complex[58] [59]. While discovered with Taq DNA polymerase, a member of the DNA polymerase sequence family A, in further studies[60] this concept to increase mismatch extension selectivity could be transferred to the B family Pfu DNA polymerase. The respective amino acids are found in the highly conserved motifs YGDTD and KXY in eukaryotes, bacteria, archaea, and viruses[61] [62].

Here we present a systematic study of the influence on the mismatch extension selectivity of all basic amino acids (arginines and lysines), which are in direct contact with the phosphate backbone of the primer strand in the closed conformation of the Klenow fragment of Taq DNA polymerase (*KlenTaq*). The chosen mutation sites vary in distance from the enzyme's active center. We identified several discriminative active mutants that were functionally characterized and evaluated in ASA, HLA typing and MSP.

## Materials and Methods

### Reagents and Instruments

Oligonucleotides were purchased from Biomers or Metabion, HeLa genomic DNA and Taq 2x master mix was bought from New England Biolabs, dNTPs were either from Roche or Fermentas, Phusion DNA polymerase was purchased from Thermo Scientific, Platinum Taq and AmpliTaq Gold DNA polymerases from Life Technologies, DNase I, SphI, and HindIII from Fermentas, the Gel Extraction and EpiTect MSP kit from Qiagen and used according to their manuals. *KlenTaq* and its respective mutants were recombinantly expressed in *E. coli* BL21 (DE3) and purified with Ni-IDA as previously described[63]. Enzyme purity and quantity were determined by SDS-PAGE using an albumin standard dilution curve. *KlenTaq* variants were stored in 50 mM Tris-HCl (pH 9.2), 16 mM (NH_4_)_2_SO_4_, 0.1% Tween20, 2.5 mM MgCl_2,_ 50% glycerol at −20°C. For real-time PCR a Chromo4 instrument from Bio-Rad or a Roche LightCycler 96 or 480 system was used. SYBR green I was purchased from Fluka. PCR was performed in the GeneAmp PCR System 9700 from Applied Biosystems. Genomic DNA samples were bought from NIBSC (Prothrombin Mutation G20210A, Human gDNA, 1st International Genetic Reference Panel 2005 - WHO International Standard or Reference Reagent Product Number 05/130).

### Saturation mutagenesis and library construction

*KlenTaq* wild-type DNA genes were amplified by PCR using Phusion DNA polymerase in the supplied buffer according to the manual. For saturation mutagenesis either NNK (forward) or MNN (reverse) primers were used. One of the following randomization primers was used together with the respective forward or reverse primer bearing a restriction site for cleavage: SphI-for [200 nM, 34 nt, 5′-d(CAT ACG GAT CCG CAT GCA GCC CTG GAG GAG GCC C)-3′] and HindIII-rev [200 nM, 36 nt, 5′-d(GCT CAG CTA ATT AAG CTT TCT CCT TGG CGG AGA GCC)-3′]. R487deg [200 nM, 61 nt, 5′-d(CTT CCG CCT GGC CGG CCA CCC CTT CAA CCT CAA CTC CNN KGA CCA GCT GGA AAG GGT CCT C) -3′], K508deg [200 nM, 45 nt, 5′-d(CCG CCA TCG GCA AGA CGG AGN NKA CCG GCA AGC GCT CCA CCA GCG) -3′], R536deg [200 nM, 50 nt, 5′-d(GTG GAG AAG ATC CTG CAG TAC NNK GAG CTC ACC AAG CTG AAG AGC ACC TA) -3′], R587deg [200 nM, 50 nt, 5′-d(CCC AAC CTC CAG AAC ATC CCC GTC NNK ACC CCG CTT GGG CAG AGG ATC CG)-3′] or R660deg [200 nM, 49 nt, 5′-d(CCC CGA AGT TGA TGG TCT TGG CCG CMN NGC GCA TCA GGG GGT CCA CGG C)-3′]. The target PCR product was purified by gel electrophoresis and used as a primer in a second PCR with either the SphI-for or HindIII-rev primer to generate the *KlenTaq* full-length product. The amplified products were purified by gel electrophoresis, digested with the respective restriction enzymes SphI and HindIII and cloned into a suitable expression vector. The ligation reaction was transformed into *E. coli* BL21 (DE3) cells and colonies were picked randomly.

### Screening and real-time ASA assay with *KlenTaq* wild-type and mutants (lysate or purified enzyme)

Reaction mixtures contained 50 mM Tris-HCl (pH 9.2), 16 mM (NH_4_)_2_SO_4_, 0.1% Tween20, 2.5 mM MgCl_2_, 250 µM of each dNTP and 0.6x SYBR Green I. As primer F20-for [750 nM, 20 nt, 5′-d(CGT TGG TCC TGA AGG AGG AT)-3′] and F20-rev [750 nM, 20 nt, 5′-d(CGC GCA GCA CGC GCC GCC GT)-3′] were used. As templates, either F90A [60 pM, 90 nt, 5′-d(CCG TCA GCT GTG CCG TCG CGC AGC ACG CGC CGC CGT GGA CAG AGG ACT GCA GAA AAT CAA CCT **A**TC CTC CTT CAG GAC CAA CGT ACA GAG)-3′] or F90G [60 pM, 90 nt, 5′-d(CCG TCA GCT GTG CCG TCG CGC AGC ACG CGC CGC CGT GGA CAG AGG ACT GCA GAA AAT CAA CCT **G**TC CTC CTT CAG GAC CAA CGT ACA GAG)-3′] were used. Both templates only differ by the SNP at the position indicated in bold and underlined letter. After an initial denaturation cycle (95°C for 3 min), the product was amplified by 30 PCR cycles (95°C for 15s, 55°C for 20 s, and 72°C for 30 s), and analyzed by melting curve measurement. *KlenTaq* wild-type and the respective mutants were used at either 50 nM or 350 nM as indicated. Lysates were prepared in reaction buffer and directly used after heat inactivation at 75°C for 40 min and subsequent centrifugation for 40 min at 4500 rpm.

### Primer extension assay with *KlenTaq* wild-type and mutants

Reaction mixtures contained 50 mM Tris·HCl (pH 9.2), 16 mM (NH_4_)_2_SO_4_, 0.1% Tween 20, 2.5 mM MgCl_2_, 250 nM DNA polymerase wild-type or mutant. DNA primer [150 nM, 20 nt, 5′-[^32^P]-d(CGT TGG TCC TGA AGG AGG A**T** )-3′], and 300 nM template F33A [300 nM, 33 nt, 5′-d(AAA TCA ACC T**A**T CCT CCT TCA GGA CCA ACG TAC)-3′] or F33G [300 nM, 33 nt, 5′-d(AAA TCA ACC T**G**T CCT CCT TCA GGA CCA ACG TAC)-3′] were used for the match or mismatch case, respectively. After an initial denaturation and annealing step (95°C for 2 min, 0.5°C/s cooling to 40°C for 30 sec), a temperature of 55.0°C was applied and the reaction was started by addition of 200 µM dNTPs. After 10 min of incubation the reactions were stopped by addition of two volumes of gel loading buffer (80% formamide, 20 mM EDTA). Product mixtures were separated by 12% denaturating PAGE and visualised using a Phosphorimager.

### Reaction kinetics of *KlenTaq* wild-type and mutants

Reaction mixtures contained 20 mM Tris·HCl (pH 7.5), 50 mM NaCl, 2.0 mM MgCl_2_, 1 µM DNA polymerase wild-type or mutant, DNA primer [100 nM, 20 nt, 5′-[^32^P]-d(CGT TGG TCC TGA AGG AGG A**T** )-3′], template F33A [130 nM, 33 nt, 5′-d(AAA TCA ACC T**A**T CCT CCT TCA GGA CCA ACG TAC)-3′] or F33G [130 nM, 33 nt, 5′-d(AAA TCA ACC T**G**T CCT CCT TCA GGA CCA ACG TAC)-3′] for the match or mismatch case, respectively. After an initial denaturation and annealing step (95°C for 2 min, 0.5°C/s cooling to 40°C for 30 sec), a temperature of 37.0°C was applied and the reaction was started by the addition of 600 µM dATP. After incubation for specified times the reactions were either stopped manually or with a KinTek Rapid Quench-Flow instrument by the addition of 300 mM EDTA. Product mixtures were separated by 12% denaturating PAGE and visualised using phosphorimaging. Quantification was done using Quantity One software from Biorad.

### ASA assay with *KlenTaq* variants using human genomic DNA (gDNA)

Reaction mixtures (20 µL) contained 50 mM Tris-HCl (pH 9.2), 16 mM (NH_4_)_2_SO_4_, 0.1% Tween20, 2.5 mM MgCl_2_, 200 µM of each dNTP, 0.6x SYBR green I, 5% DMSO, 20 ng HeLa gDNA, and 100 nM of the respective *KlenTaq* variant. As a forward primer either F2forG [100 nM, 23 nt, 5′-d(CCC AAT AAA AGT GAC TCT CAG C**G** )-3′] or F2forA [100 nM, 23 nt, 5′-d(CCC AAT AAA AGT GAC TCT CAG C**A** )-3′] was used in conjunction with primer F2rev [100 nM, 20 nt, 5′-d(CCA GAG AGC TGC CCA TGA AT)-3′. Real-time PCR was conducted by an initial denaturation cycle (95°C for 3 min), followed by 50 PCR cycles (95°C for 20 s, 57°C for 10 s and 72°C for 30 s) and analysis of an amplicon size of 64 base-pairs by melting curve measurement.

### ASA assay with *KlenTaq* R660V mutant and commercially available DNA polymerases using human gDNA allele-standards

Reaction mixtures (10 µL) contained 50 mM Tris-HCl (pH 9.2), 16 mM (NH_4_)_2_SO_4_, 0.1% Tween20, 2.0 mM MgCl_2_, 200 µM of each dNTP, 1x SYBR green I, 10 ng gDNA allele-standard and 100 nM *KlenTaq* R660V. As forward primer F2forG [100 nM, 23 nt, 5′-d(CCC AAT AAA AGT GAC TCT CAG C**G** )-3′] or F2forA [100 nM, 23 nt, 5′-d(CCC AAT AAA AGT GAC TCT CAG C**A** )-3′] was used in conjunction with reverse primer F2rev [100 nM, 20 nt, 5′-d(CCA GAG AGC TGC CCA TGA AT)-3′]. Taq 2X Master Mix (New England Biolabs) and Platinum Taq (Life Technologies) were used as described by the manufacturer. In case of the Platinum Taq, the reaction contained 0.5 U enzyme and 1.5 mM MgCl_2_. Templates for homozygote, heterozygote and wild type were used. After an initial denaturation cycle (95°C for 3 min), the product was amplified by 60 PCR cycles (95°C for 20 s, 57°C for 10 s, and 72°C for 30 s), and analyzed by melting curve measurement. For endpoint PCRs, the cycling was stopped after 50 cycles and the PCR product immediately analysed by 2.5% agarose gel electrophoresis.

### Multiplexing ASA assay with R660V from gDNA allele-standards and blood

SNP Factor II G20210A (respective amplicon size is 64 bp for the A-allele or 94 bp for the G-allele) was detected by direct amplification from 10 ng of genomic reference DNA using both allele-specific primers in the same reaction: F2forG [100 nM, 53 nt, 5′-d(ATC CAA CTC TCT ACG CAA TGG CAC TAG AGA CCC AAT AAA AGT GAC TCT CAG C**G** )-3′], F2forA [125 nM, 23 nt, 5′-d(CCC AAT AAA AGT GAC TCT CAG C**A** )-3′] and reverse primer F2rev [100 nM, 20 nt, 5′-d(CCA GAG AGC TGC CCA TGA AT)-3′]. Real-time ASA reactions contained 50 mM Tris-HCl (pH 9.2), 16 mM (NH_4_)_2_SO_4_, 0.1% Tween20, 2.5 mM MgCl_2_, 200 µM of each dNTP, 1x SYBR Green I, and 100 nM *KlenTaq* R660V. In case of a blood sample 30x SYBR Green I and 500 nM of the DNA polymerase was used. Preceding the reaction, 0.5 µl blood per well was allowed to dry at room temperature for 5 min before adding 10 µl of the above described reaction solution. After an initial denaturation cycle (95°C for 3 min), the product was amplified by 40 PCR cycles (95°C for 15 s, 57°C for 5 s, and 72°C for 15 s), and immediately analysed by melting curve measurement.

### HLA typing with *KlenTaq* R660V and AmpliTaq Gold DNA polymerase

A primer mix specific for most alleles of the HLA-DRB1*03, 11, 13, and 14 group (80) with a sense primer located in intron 1 I1-RB9 [100 nM, 17 nt, 5′-d(TGG TGG GCG TTG GGG GG)-3′] and an antisense primer located in intron 2 I2-RB28 [100 nM, 21 nt, 5′-d(ACA CAC ACA CTC AGA TTC CCA)-3′], yielding a 465 bp product were used in a PCR mixture consisting of 50 ng genomic DNA, 50 mM Tris-HCl, pH 8.0; 10 mM KCl, 1.5 mM MgCl_2_, 7.5 mM (NH_4_)_2_SO_4_, 200 µM of each dNTP, and 1 U of AmpliTaq Gold DNA polymerase or 100 nM *KlenTaq* R660V, respectively. PCR amplifications were carried out in a GeneAmp PCR System 9700 (Applied Biosystems). After an initial denaturation step at 94°C for 10 min, samples were subjected to 10 two-temperature cycles, each consisting of denaturation at 94°C for 30 s, annealing and extension at 65°C for 50 s, followed by 20 three-temperature cycles with a decreased annealing temperature of 62°C and an extension at 72°C for 30 s. For visualization, 8 µl of the amplification product were analysed by 2% agarose gel electrophoresis prestained with ethidium bromide (0.2 mg/ml).

### Error rate determination of *KlenTaq* R660V mutant

We used a PCR-based assay, reported by Patel *et al*., to determine the error rate and the error spectrum[64]. PCR reactions (100 µl) contained 30 fmol template (pGDR11-codon optimized gene encoding *KlenTaq* wild-type[63]), 250 µM of each dNTP, forward primer [400 nM, 40-nt, 5′-d(GGA TCC GCA TGC AGC ACT GGA AGA AGC ACC TTG GCC TCC G)-3′] and reverse pimer [400 nM, 32 nt, 5′-d(CTA ATT AAG CTT TTA TTC TTT TGC AGA CAG CC)-3′], 80 nM of DNA polymerase in 50 mM Tris-HCl (pH 9.2), 16 mM (NH_4_)_2_SO_4_, 0.1% Tween 20, 2.5 mM MgCl_2_. After an initial denaturation cycle (95°C for 1 min), the product was amplified by 30 PCR cycles (95°C for 60 s, 65.1°C for 60 s and 72°C for 2 min). PCR yield was determined by agarose gel analysis to calculate the number of replication cycles. PCR products were subsequently purified using preparative gel electrophoresis, digested with SphI and HindIII and cloned into the expression vector. Colonies were picked randomly and sequenced at GATC Biotech. Sequences were analyzed with SEQUENCHER™ 5.0.1 software. The number of mutations per bp per clone yielded in the mutation frequency. The error-rate was determined by the mutation frequency divided by the number of replication cycles.

### Real-time MSP assay with *KlenTaq* R660V mutant

Reaction mixtures contained 50 mM Tris-HCl (pH 9.2), 16 mM (NH_4_)_2_SO_4_, 0.1% Tween20, 2.0 mM MgCl_2_, 200 µM of each dNTP, 1x SYBR green I and 50 nM *KlenTaq* R660V. As forward primer MSP-Forw [100 nM, 30 nt, 5′-d(GCG CGA TTC GTT GTT TAT TAG TTA TTA TGT)-3′] was used in conjunction with reverse primers MSP-Rev-G [100 nM, 31 nt, 5′-d(TCG AAA TCC GAA ATA ATC CCA TCC AAC TAC **G** )-3′] or MSP-Rev-A [100 nM, 31 nt, 5′-d(TCG AAA TCC GAA ATA ATC CCA TCC AAC TAC **A** )-3′]. As templates, either Temp Sept9-5 mC [150 pM, 85 nt, 5′-d(TTC GCG CGA TTC GTT GTT TAT TAG TTA TTA TGT CGG ATT TCG CGG TTA ACG **C^Me^**GT AGT TGG ATG GGA TTA TTT CGG ATT TCG AAG G)-3′] or Sept9-U [150 pM, 85 nt, 5′-d(TTC GCG CGA TTC GTT GTT TAT TAG TTA TTA TGT CGG ATT TCG CGG TTA ACG **U**GT AGT TGG ATG GGA TTA TTT CGG ATT TCG AAG G)-3′] were used. After an initial denaturation cycle (95°C for 3 min), the product was amplified by 50 PCR cycles (95°C for 20 s, 57°C for 10 s and 72°C for 30 s) and analyzed by melting curve measurement. Qiagen EpiTect MSP kit was used as described in the provided manual.

## Results and Discussion

**Figure 1:**
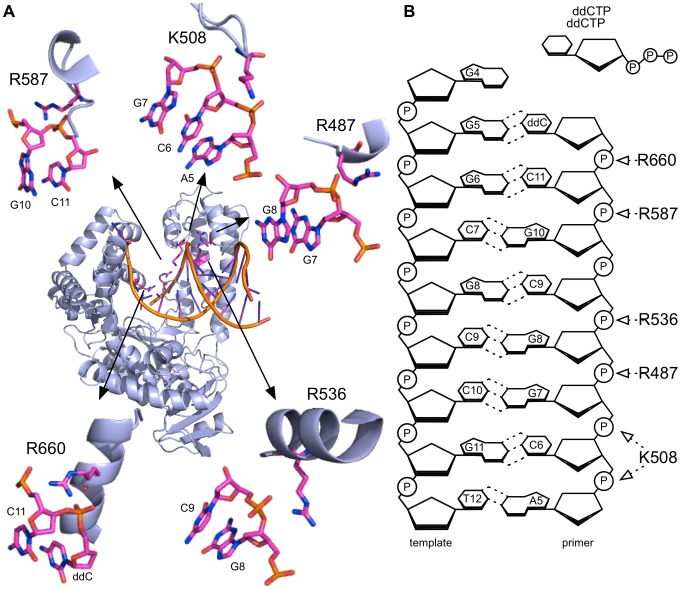
Investigated mutation sites in *KlenTaq*. A) Crystal structure view showing the active ternary complex of *KlenTaq*. The enlarged sections show the interaction of the amino acid of interest with the phosphate of the primer backbone (PDB-code 3KTQ[68]). B) 2D-schematic drawing of the DNA substrate, template (left) and primer (right). Arrows indicate the interaction of the amino acids according to [68].

For our studies we employed *KlenTaq* [65]. Compared to Taq DNA polymerase, *KlenTaq* is approximately twice as thermostable and only displays half the error rate of *Taq* DNA polymerase[65] [66] [67]. Structural data of *KlenTaq* shows that in the closed conformation five basic amino acids make contact with the primer strand[68]. Those are K508, R487, R536, R587 and R660 (see Figure 1). In order to study the impact of these basic amino acids on selectivity and activity we used saturation mutagenesis at these positions. Using degenerate NNK-primers for each amino acid position, a library consisting of at least 320 mutants was compiled and screened, guaranteeing a 99.9% coverage of all 20 possible amino acids under ideal mutagenesis conditions[69].

Screening was conducted as described previously in a high-throughput format using heat-treated *E. coli* lysates[70]. Our screening strategy uses 384 well plate libraries and monitors DNA polymerase activity by *SYBR green* I mediated quantification of synthesized double stranded DNA and its subsequent melting temperature assessment. Each mutant was hence tested in parallel and real-time for: a) thermostability for sufficient PCR activity, b) specificity by producing the correct amplicon length and c) their 3′ end mismatch extension discrimination. One reaction contained a matched primer template duplex whereas in another reaction a single mismatch at the 3′-terminus of the primer template complex was induced through exchange of one nucleotide in the template strand (A into G). Entities with increased extension selectivity were identified as those variants causing amplification curves with lower threshold crossing (c(t)) cycle numbers with matched DNA substrates than with mismatched DNA substrates. Depending on the position of the respective arginine or lysine residue, mutagenesis led to varying amounts of PCR inactive mutants. For positions K508 and R587, both located in loops (Figure 1), more than 65% of all mutants were PCR active. When mutating R536, located in an alpha helix, only 12% of the mutants were PCR active. Mutation of position R487, located between a loop and an alpha helix, caused 30% PCR active mutants. Surprisingly substitution of R660 resulted in over 60% of PCR active mutants although it is located in an alpha helix. Interestingly, we identified variants with increased extension selectivity for all five investigated positions (R487, K508, R536, R587 and R660, see Figure 1). In order to exclude expression level variations and other artefacts from the crude heat-treated *E. coli* lysates, we verified our findings by purifying selected hits and repeating the ASA assay. While the wild-type enzyme displays no significant discrimination under conditions used in the screening, employment of all selected mutants leads to significantly increased mismatch extension selectivity. The most promising examples for each position were sequenced. In only three cases among the identified hits was the basic amino acid arginine exchanged to another basic amino acid such as histidine (R487H) or lysine (R536K and R587K). Two variants with mutations to tyrosine and threonine were found (K508Y and R660T). In several cases substitutions towards hydrophobic amino acids were identified: R487V, K508W, R587I, and R660V. In fact, these results are in agreement with our earlier findings showing that mutations towards hydrophobic amino acids can increase mismatch extension selectivity[60] [55] [71].

**Figure 2:**
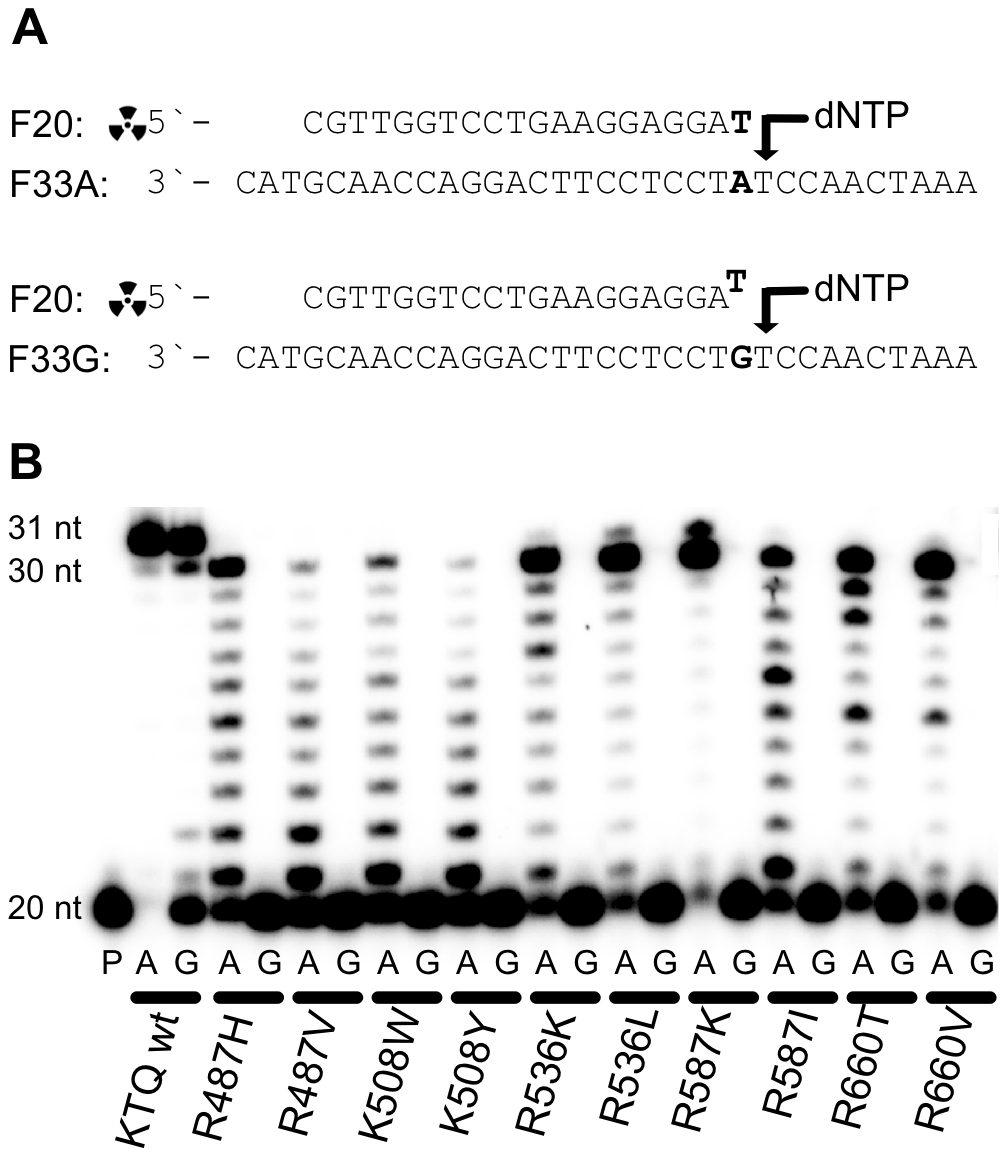
Primer extension experiments with *KlenTaq* wild-type and mutants. A) Primer and template sequence. The radioactively labelled primer is either 3’ matched or mismatched depending on the template. B) PAGE of primer extension, P  =  primer, A  =  match case template, G  =  mismatch case template. Mutants and *KlenTaq* wild-type as indicated.

In order to directly compare activity and mismatch extension selectivity of the selected mutants we performed primer extension experiments next. Therefore we used a 20 nucleotides (nt) radioactively labelled primer and two 33 nt templates having the same sequence except for one specific position (Figure 2A). Depending on the template, the 3’-end of the primer was either matched or mismatched. Reactions were incubated isothermally at 55°C and started by addition of dNTPs and quenched after 10 min, followed by PAGE analysis. As depicted in Figure 2 the *KlenTaq* wild-type shows full-length product formation and, as reported before for 3’-5’- exonuclease-deficient polymerases, a non-templated additional nucleotide incorporation for the match and the mismatch case[72] [73] [74]. Notably not all primer is converted for the mismatch case indicating the intrinsic mismatch extension discrimination. None of the mutants show product formation for the mismatch case after 10 min, but yield full-length product for the match case. Whereas some mutants like R487V or K508Y clearly show reduced activity, which is detected by shorter reaction products, other mutants such as R587K or R660V show clean full-length product formation in the match case indicating a conserved high activity comparable to the wild-type enzyme.

**Figure 3:**
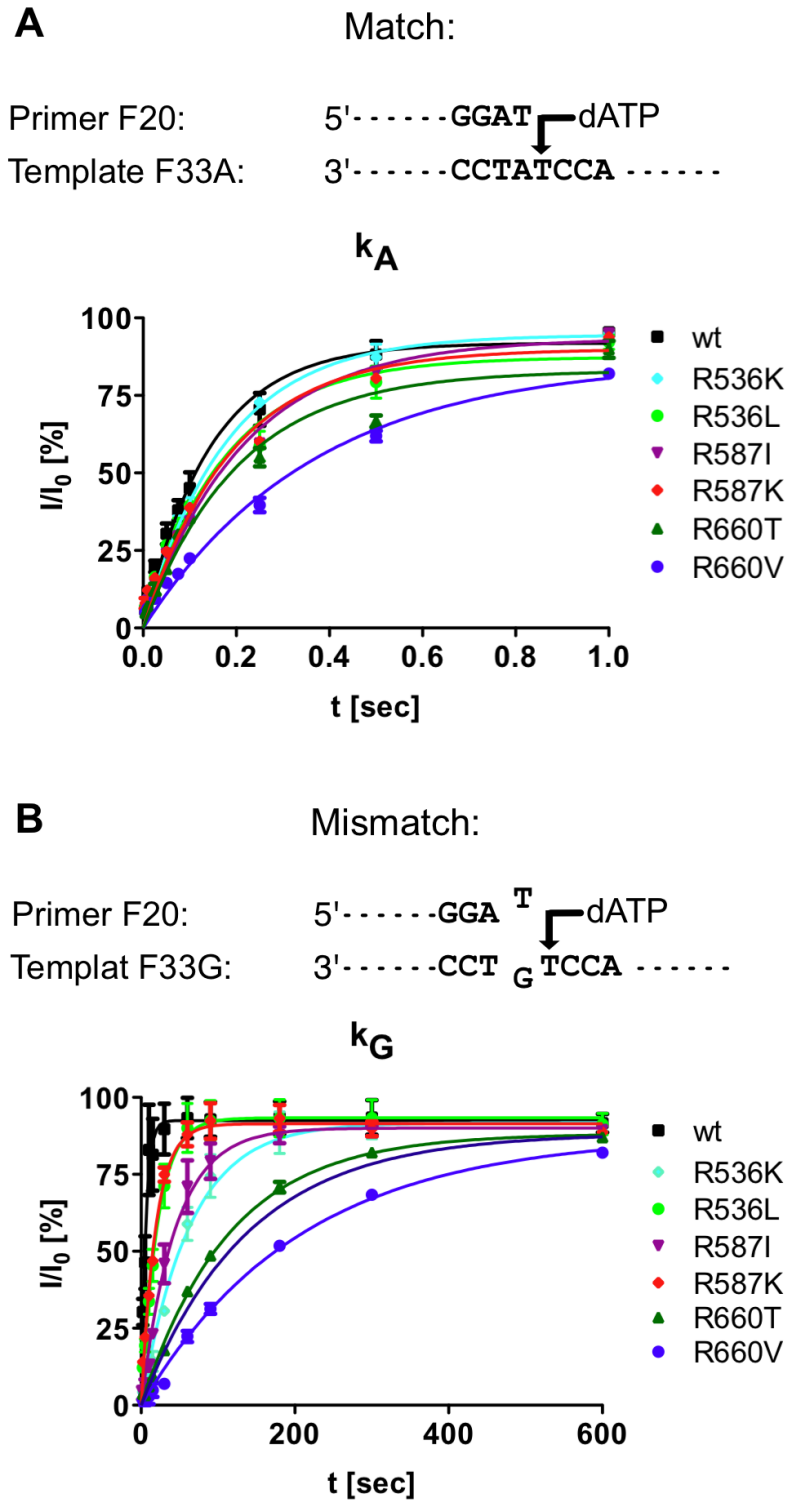
Single turnover rates (pre-steady-state). A) Conversion of primer vs. time for the match case. B) Conversion of primer vs. time for the mismatch case.

As mutations at position R487 and K508 reduced the activity significantly (see Figure 2), they were excluded from further analyses. To characterise the identified mutants in more detail, we investigated single nucleotide incorporation turnover rates (under pre-steady state conditions) for extension from matched and mismatched primer strands. High concentrations of enzyme were used to saturate binding of the DNA substrate. At high dNTP concentrations (600 µM) the first order rate reflects k_cat_ [75]. The results are shown in Figure 3 and Table 1. Notably, reaction times for the mismatch cases were up to 600 s whereas for the match cases full conversion was detected after 1 s for all polymerases.

**Table 1:** Single turnover rates with std. error (pre-steady-state) of *KlenTaq* wild-type (wt) and mutants.

	k_A_ (s^−1^)^a^	k_G_ (s^−1^)^b^	k_A_/k_G_	(k_A_/k_G_)/(kA/kG)wt
**wild-type**	7.00±0.59	0.17±0.02	41,2	1.00
**R487H**	4.74±0.26	0.0062±0.0003	766	18.6
**R487V**	3.06±0.29	0.0021±0.0001	1485	36.0
**K508W**	3.65±0.22	0.0042±0.0003	878	21.3
**K508Y**	5.01±0.37	0.0084±0.0004	595	14.4
**R536K**	5.65±0.28	0.016±0.001	355	8.61
**R536L**	5.69±0.69	0.047±0.004	121	2.94
**R587I**	4.66 ± 0.32	0.023±0.002	205	4.98
**R587K**	5.29±0.48	0.053±0.003	100	2.44
**R660T**	4.90±0.55	0.0088±0.0003	559	13.6
**R660V**	2.72±0.25	0.0093±0.0004	294	7.13

a k_A_  =  k_cat_ match primer in conversions per second (s^−1^)

b k_G_  =  k_cat_ mismatch primer in conversions per second (s^−1^)

A measure for the selectivity is k_A_/k_G_ as shown in Figure 3 and Table 1. This ratio is significantly increased in the selected mutants, compared to the wild-type enzyme, given as (k_A_/k_G_)/(k_A_/k_G_)wt (wt  =  wild-type). For instance, the discrimination of the matched vs. mismatched primer was increased by up to 36-fold in mutant R487V compared to *KlenTaq* wild-type.

Attempting to further improve the mismatch discrimination selectivity, we applied a shuffling approach, as this has been reported to be promising for other directed evolution approaches in order to optimize *KlenTaq* properties[76]. Therefore, we shuffled the two most interesting mutant genes for each position and the wild-type gene and created a new library consisting of over 2300 members. Under ideal shuffling conditions, this covers all 243 possible mutant combinations with over 98% completeness[77]. This library was screened as described above; however except for some single mutants, the most interesting hit we could isolate contained the following mutations K508Y, R587K, and R660T. Experiments with the purified enzyme showed similar discrimination as the best single mutants, but lower PCR activity. Sequencing of inactive variants or variants with poor activity revealed that the combination of some of the best single point mutations leads to a drop in activity. Since some of these variants showed a further loss in activity during purification and storage, we assume that the combination of these mutations leads to a decrease in protein stability. Noteworthy, none of the above-described single mutants showed any loss in PCR activity, during purification or long-term storage over six months and more at −20°C.

Next, we investigated the performance of the most active single mutants on human HeLa genomic DNA in the context of the Factor II prothrombin SNP. The prothrombin SNP is connected to an increased thrombosis risk and therefore of medical interest[10]. All mutants show increased Δc(t) values on genomic DNA compared to *KlenTaq* wild-type. Most mutants show wild-type like high activity with c(t) values of 30 for the match case compared to *KlenTaq* wild-type with a c(t) value of 28 (Table 2). The mutant R660V shows the highest mismatch extension discrimination of 18 cycles compared to 8.5 for the *KlenTaq* wild-type as well as wild-type like high activity. Therefore, further experiments were carried out with this enzyme.

**Table 2:** Threshold crossing points (c(t)) of real-time PCR experiments, detecting SNP Factor II on HeLa genomic DNA.

	wild-type	R536K	R536L	R587I	R587K	R660T	R660V
**c (t) match**	28	30	30	32	31	30	30
**c (t) mismatch**	37	44	46	49	47	46	48
**Δc(t)**	8.5	13	16	17	15	16	18

**Figure 4:**
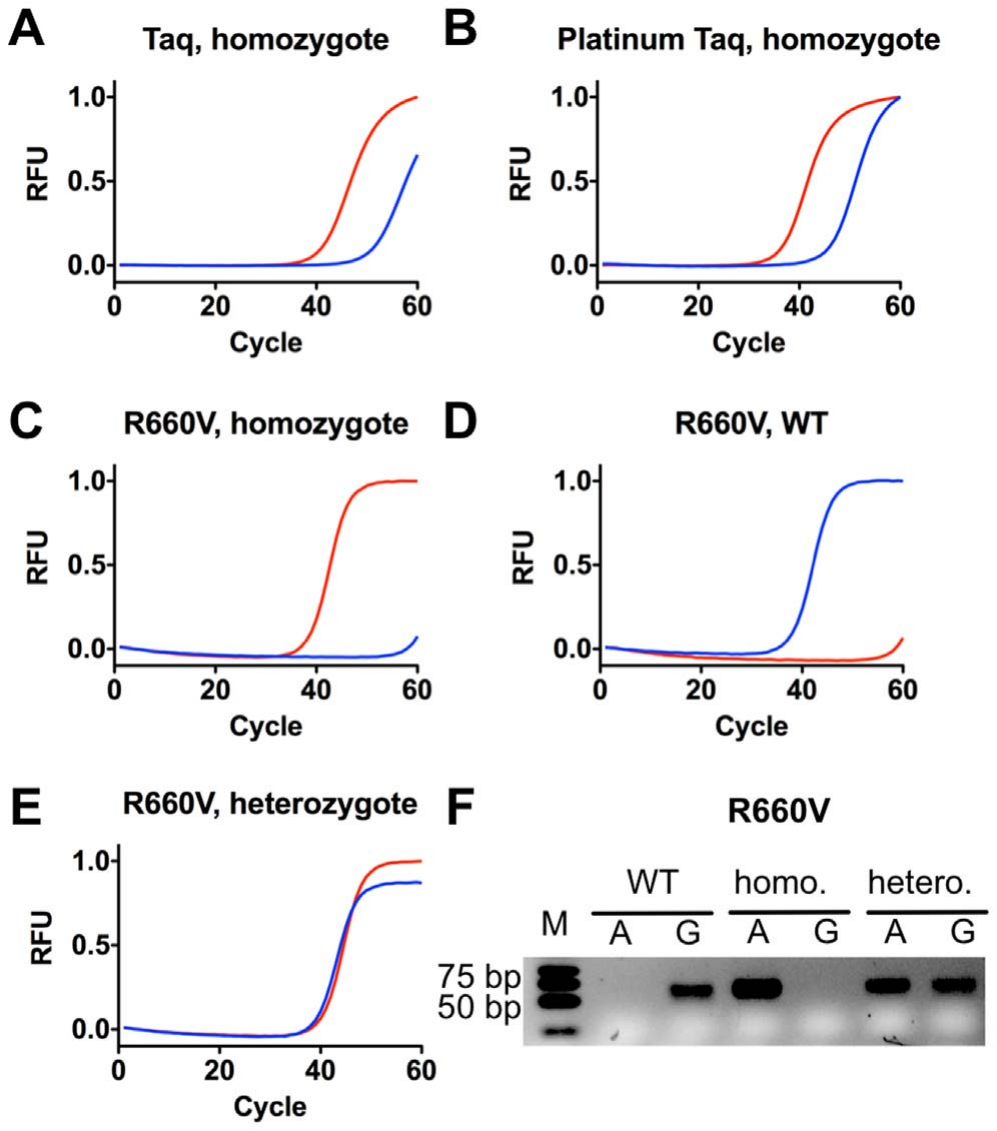
ASA assay of Factor II prothrombin, Human gDNA (WHO International Standards) with R660V mutant. Allele-specific primers A and G result in a 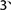 end match or mismatch depending on the chosen template. In red primer A is shown, in blue primer G. RFU  =  relative fluorescence units. A) ASA real time PCR curves of Factor II homogygote (genotype A/A) using Taq DNA polymerase, B) Platinum Taq DNA polymerase, or C) mutant R660V. D) ASA real time PCR curves of Factor II wild-type (genotype G/G), E) ASA real time PCR curves of Factor II heterozygote (genotype G/A), F) Agarose gel electrophoresis analysis of ASA PCR. M  =  Marker, A  =  Primer A, G  =  Primer G, wild-type (WT) indicates the used wild-type template, homo, the homozygote and hetero, the heterozygote template.

First, we evaluated how mutant R660V performs in comparison with commercially available polymerases. Thus, we tested Platinum Taq DNA polymerase, a commercially available wild-type Taq DNA polymerase in comparison to mutant R660V (see Figure 4, A-C) in their capability to amplify from a homozygote (A/A) human gDNA. The gDNA was obtained from the National Institute for Biological Standards and Control (NIBSC). We found, that the use of mutant R660V is resulting in almost doubled Δc(t) values compared to the commercially available enzymes. Encouraged by this result, we also tested the remaining naturally occurring cases: wild-type (genotype G/G) and heterozygote (genotype G/A) with the mutant R660V (see Figure 4, D-F).

As shown in Figure 4 C-D, for both homozygote cases product formation was only detected for the corresponding match primer. The signal of the mismatched primers is shifted to higher c(t) values with the start of the exponential phase after 60 cycles. In the case of the heterozygote template product formation was detected for both allele specific primers, as expected. Therefore, fast and reliable SNP genotyping is possible and with a higher reliability compared to the commercially available polymerases standardly used. Detection is also possible by standard PCR and subsequent analyses by agarose gel electrophoresis (Figure 4 F). After 50 PCR cycles clear product bands with the expected migration in the gel are visible. As shown all three possible SNP genotypes can be clearly distinguished.

**Figure 5:**
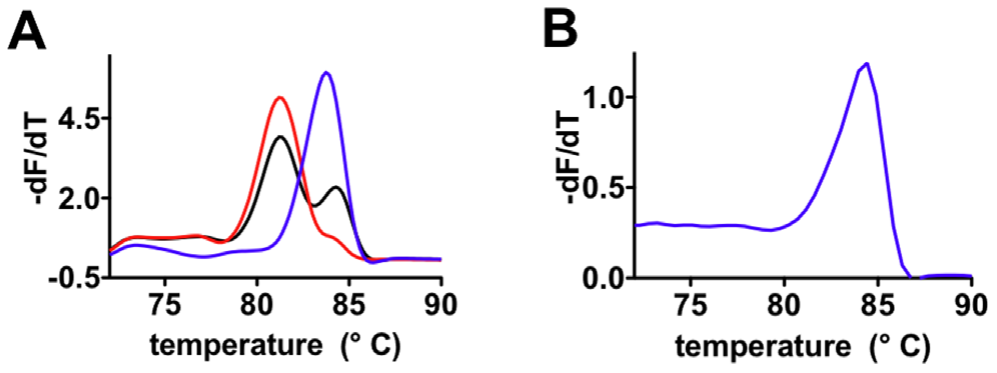
Multiplexing ASA melting curve assay of Factor II prothrombin. A) Melting curves of the multiplexing ASA products resulting from three different reference gDNA samples are shown (wild-type in blue (genotype G/G), heterozygote in black (genotype G/A), homozygote in red (genotype A/A)). B) Resulting melting curve from a whole blood sample, identifying the wild-type genotype.

Next we established a multiplexing ASA assay for the Factor II prothrombin SNP by incorporating a 30-nucleotide overhanging sequence at the 5′end of one of the allele-specific primers. Thus, both alleles can be detected in the same reaction according to their respective melting temperatures[78]. We successfully tested our assay on all three gDNA references, displaying the wild-type, homozygote or heterozygote genotype (see Figure 5A). Notably, all experiments can be conducted in any suitable real-time PCR machine able to perform melting point analysis with a suitable dye such as SYBR Green I.

As reported previously, Taq DNA polymerase activity is significantly inhibited in the presence of less than 0.2% whole blood[79] [80]. In contrast, *KlenTaq* exhibits an increased resistance[80]. Thus, we studied PCR performance of mutant R660V in the presence of whole blood. Due to fluorescence quenching effects of blood on SYBR Green I[80], a 30-fold increased dye concentration was required for real-time PCR. Additionally we used a 5-times higher DNA polymerase concentration in comparison to ASA using purified gDNA samples. The genotype of the blood sample was successfully identified as wild-type (wt; G/G), as a melting peak was observed only for the G-allelic primer, indicating that the direct detection of SNPs from whole blood samples is achievable (Figure 5B). This may pave the way for clinical diagnostics without the need of time and cost intensive sample preparations.

**Figure 6:**
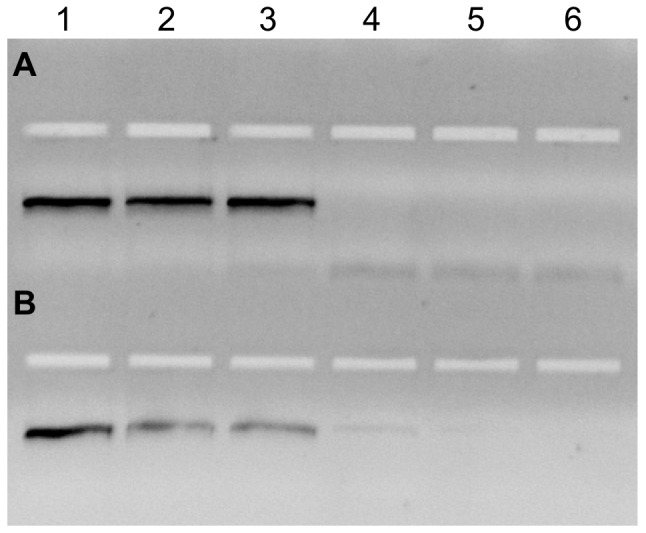
HLA-PCR SBT with R660V and AmpliTaq Gold. PCR amplification of six DNAs using either *KlenTaq* R660V (A) or AmpliTaq Gold (B). The HLA-DRB1 profiles of the genomic DNA are: lane 1: HLA-DRB1* 13∶01∶01, 13∶02∶01; lane 2: HLA-DRB1* 03∶01∶01, 11∶01∶01; lane 3: HLA-DRB1 01∶01∶01, 13∶01∶01; lane 4: HLA-DRB1* 08∶01∶03, 08∶01∶03; lane 5: HLA-DRB1* 01∶01∶01, 07∶01∶01; lane 6: HLA-DRB1* 07∶01∶01, 15∶01∶01.

Subsequently we examined the performance of *KlenTaq* R660V in HLA typing by sequence-specific primed PCR (PCR-SSP). A primer mix specific for a limited set of HLA alleles from the HLA-DRB1 allelic groups HLA-DRB1*03. *11. *13. *14 has been used to compare AmpliTaq Gold DNA polymerase and *KlenTaq* R660V with regard to sensitivity and specificity under the PCR conditions previously described[81]. The specificity of the primer mix for the HLA-DRB1 allelic groups HLA-DRB1*03, *11, *13, *14 relies on a two base pair difference at the 3′ end of the sense primer in intron 1 (I1-RB9). The target sequence of the antisense primer in intron 2 (I2-RB28) is identical in all HLA-DRB1 alleles tested. The reaction is designed in a way that the employed primer mix should amplify a 465 bp product of the DNAs in the setups analyzed in rows 1, 2 and 3, but not in rows 4, 5 and 6 of Figure 6. We found, that while *KlenTaq* R660V gave clear results in the sensitivity as well as the specificity controls, AmpliTaq Gold DNA polymerase also led to weak amplifications in rows 4 and 5 demonstrating less specificity. The designation of the genes and alleles listed in Figure 6 follow the WHO Nomenclature for Factors of the HLA system (http://hla.alleles.org/nomenclature/index.html). After the HLA-prefix followed by the name of the gene (e.g. DRB1) an asterix is inserted to separate the gene's name from the allele designation. Each HLA allele name has a unique number corresponding to up to four sets of digits separated by colons. The digits before the first colon describe the type, which often corresponds to the serological antigen carried by an allotype. The next set of digits is used to list the subtypes, numbers being assigned in the order in which DNA sequences have been determined. Alleles whose numbers differ in the two sets of digits must differ in one or more nucleotide substitutions that change the amino acid sequence of the encoded protein. Alleles that differ only by synonymous nucleotide substitutions (also called silent or non-coding substitutions) within the coding sequence are distinguished by the use of the third set of digits. Alleles that only differ by sequence polymorphisms in the introns or in the 5′ or 3′ untranslated regions that flank the exons and introns are distinguished by the use of a fourth set of digits. The primer mix used in figure 6 is specific for all alleles of the HLA-DRB1 types (digits before the first colon) 03, 11, 13 and 14. All other alleles should not be amplified.

We next used a PCR-based assay to determine the error rate and the error spectrum of *KlenTaq* R660V[64]. A 1657 base pairs DNA fragment was amplified by PCR and cloned into a suitable expression vector, transformed into *E. coli* cells and several single clones analysed by sequencing. With an error rate of 2.8×10^−5^ mutations per base per duplication *KlenTaq* R660V exhibits somewhat improved fidelity than *KlenTaq* wild-type with 8.8×10^−5^ mutations per base per division (Table 3) [63]. Notably, mainly the number of transitions dropped whereas the number of transversions is unaffected.

**Table 3:** Error rate of *KlenTaq* wild-type and *KlenTaq* R660V.

Enzyme	No. of clones	Average no. of mutations per clone^a^	Error rate^b^ [10<sup>−5</sup>]	No. of individual substitutions						No. of deletions	No. of insertions
				Transitions		Transversions					
				AT → GC	GC → AT	AT → TA	AT → CG	GC → TA	GC → CG		
KTQ wt^c^	32	0.4	8.8	5	4	0	0	1	0	3	0
R660V	26	0.2	2.8	2	0	2	0	1	0	0	0

a Number of mutations per 650 bases (*KlenTaq* wild-type) or 726 bases (R660V) sequenced per clone.

b Error rate equals number of mutations per base per division.

c Data taken from [63].

**Figure 7:**
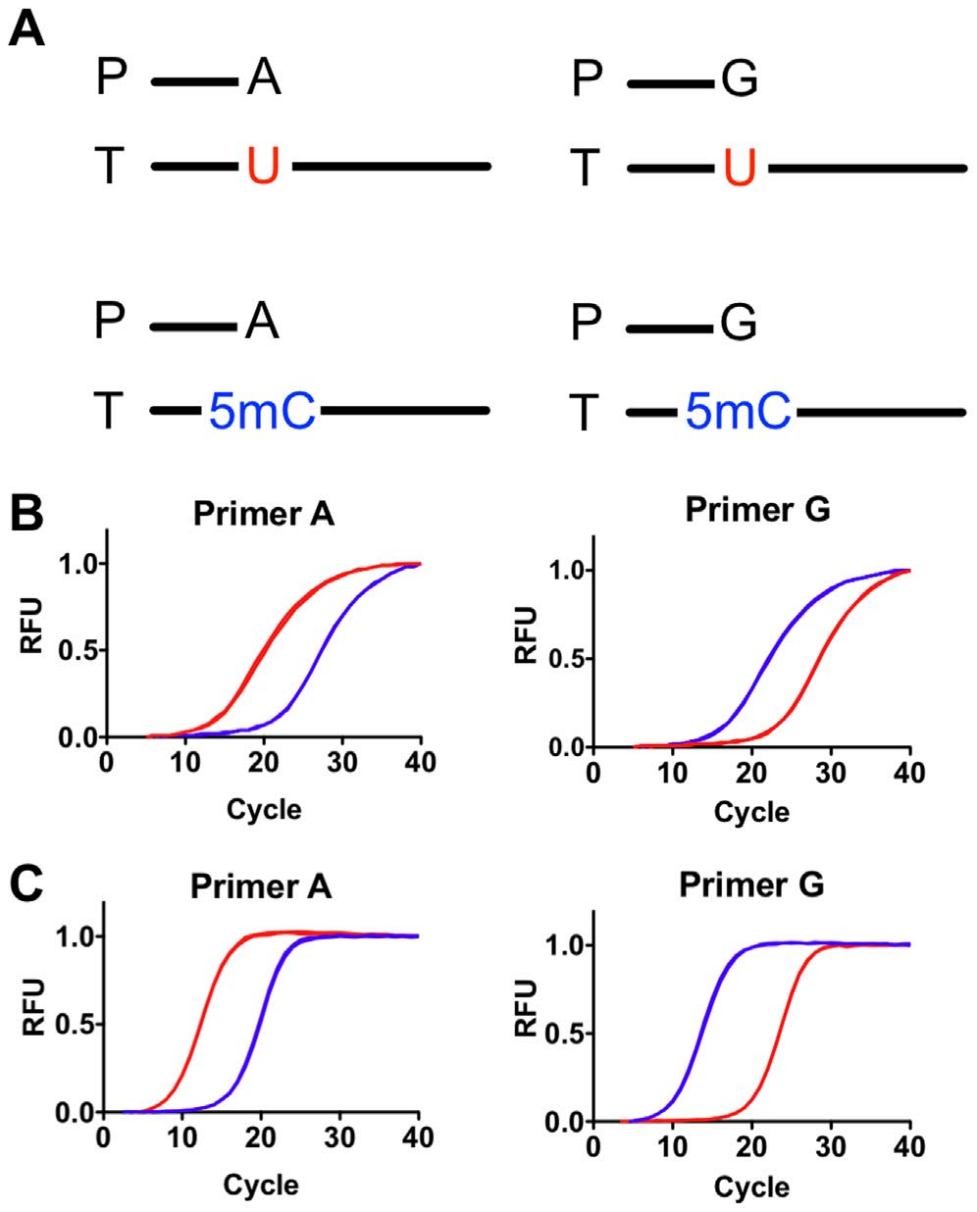
Real-time PCR spectra of MSP with mutant R660V. As template either a template with 5(blue) or with U (red) was used. Primers A and G result in a 3’-end match or mismatch depending on the chosen template. RFU  =  relative fluorescence units. A) Graphic depiction of used primer (P) and template (T) setup, B) Real-time PCR curves using a commercially available kit. Template with 5 mC (blue) or with U (red) and indicated primers were used. C) Real-time PCR curves for *KlenTaq* R660V. Template with 5 mC (blue) or with U (red) and indicated primers were used.

Finally, we explored the suitability of KlenTaq R660V for 5 mC detection and performed MSP within the genomic sequence context of Septin 9. Hypermethylation in the promoter region of the Septin 9 gene is a known colon cancer marker [82]. In these experiments we employed two templates that only differ at the position that is opposite the 3′-primer end. The primer terminates either opposite 5 mC or dU, the product after bisulfite treatment (Figure 7A). To benchmark the performance of *KlenTaq* R660V we also used a commercially available kit with the same templates and primers. As shown in Figure 7 *KlenTaq* R660V exhibits clear discrimination for both used primers with the respective mismatch template as well as high activity (Figure 7). Compared with the commercial kit, mutant *KlenTaq* R660V shows higher activity and discrimination. Thus, robust and fast detection of the epigenetic methylation status is possible using *KlenTaq* R660V.

## Conclusions

Here we demonstrated that the selectivity of a *KlenTaq* DNA polymerase can be altered by substituting a polar amino acid residue that interacts with the backbone of the primer strand. We successfully identified mutants with increased mismatch selectivity for each examined amino acid position. These findings emphasise the power of combining an initial rational design approach with the rigorous use of a screening based combinatorial enzyme design. However, by gene shuffling the best performing single mutants, we were not able to further improve the desired properties, as combinations of advantageous single point mutations resulted in decreased protein stability and activity. The most promising mutant was thoroughly characterized. We selected the mutant R660V for investigation and found that the enzyme has increased mismatch selectivity and could be used even in multiplexing assays using genomic DNA templates demonstrating its suitability for SNP detection. Additionally, *KlenTaq* R660V is able to perform ASA from DNA in the presence of whole blood with no previous DNA purification. We could also show that *KlenTaq* R660V is suitable for application in MSP to detect the methylation status at a single site. To investigate the impact of the single mutation on overall DNA polymerase selectivity we determined the error rate and spectra of *KlenTaq* R660V and found it to be somewhat increased compared to the wild-type enzyme. Furthermore, the proficiency in HLA typing of *KlenTaq* R660V was investigated. In HLA typing by sequence-specific primed PCR (PCR-SSP) we compared *KlenTaq* R660V with the commercially available AmpliTaq Gold which is widely used in this field. The specificity of the primer mix for the HLA-DRB1 allelic groups HLA-DRB1*03, *11, *13, *14 relies on a two base pair difference at the 3′ end of the sense primer in intron 1 (I1-RB9). The target sequence of the antisense primer in intron 2 (I2-RB28) is identical in all HLA-DRB1 alleles tested. Whereas the sensitivity of the Taq DNA polymerases was equivalent the specificity of the *KlenTaq* R660V was superior.
